# Inorganic Photocatalytic Enhancement: Activated RhB Photodegradation by Surface Modification of SnO_2_ Nanocrystals with V_2_O_5_-like species

**DOI:** 10.1038/srep44763

**Published:** 2017-03-16

**Authors:** Mauro Epifani, Saulius Kaciulis, Alessio Mezzi, Davide Altamura, Cinzia Giannini, Raül Díaz, Carmen Force, Aziz Genç, Jordi Arbiol, Pietro Siciliano, Elisabetta Comini, Isabella Concina

**Affiliations:** 1 Istituto per la Microelettronica e i Microsistemi, IMM-CNR, Via Monteroni, 73100 Lecce, Italy; 2 Istituto per lo Studio dei Materiali Nanostrutturati, ISMN–CNR, PO Box 10, 00015 Monterotondo Stazione, Roma, Italy; 3 Istituto di Cristallografia, IC-CNR, Via Giovanni Amendola, 122/O, 70126 Bari, Italy; 4 Electrochemical Processes Unit, IMDEA Energy Institute, Avda. Ramón de la Sagra, 3 28935 Móstoles, Spain; 5 NMR Unit, Centro de Apoyo Tecnológico, Universidad Rey Juan Carlos, c/Tulipán, s/n, 28933 Móstoles, Spain; 6 Catalan Institute of Nanoscience and Nanotechnology (ICN2), CSIC and The Barcelona Institute of Science and Technology, Campus UAB, Bellaterra, 08193 Barcelona, Spain; 7 Department of Information Engineering, Brescia University, Via Valotti 9, 25133 Brescia, Italy; 8 CNR-INO SENSOR Lab, Via Branze 45, 25123 Brescia, Italy; 9 Luleå University of Technology, 971 98 Luleå, Sweden

## Abstract

SnO_2_ nanocrystals were prepared by precipitation in dodecylamine at 100 °C, then they were reacted with vanadium chloromethoxide in oleic acid at 250 °C. The resulting materials were heat-treated at various temperatures up to 650 °C for thermal stabilization, chemical purification and for studying the overall structural transformations. From the crossed use of various characterization techniques, it emerged that the as-prepared materials were constituted by cassiterite SnO_2_ nanocrystals with a surface modified by isolated V(IV) oxide species. After heat-treatment at 400 °C, the SnO_2_ nanocrystals were wrapped by layers composed of vanadium oxide (IV-V mixed oxidation state) and carbon residuals. After heating at 500 °C, only SnO_2_ cassiterite nanocrystals were obtained, with a mean size of 2.8 nm and wrapped by only V_2_O_5_-like species. The samples heat-treated at 500 °C were tested as RhB photodegradation catalysts. At 10^−7^ M concentration, all RhB was degraded within 1 h of reaction, at a much faster rate than all pure SnO_2_ materials reported until now.

Surface management is critical in such fields as heterogeneous catalysis, gas-sensors, photocatalysis and related applications, for obvious reasons of available reaction sites, and appears even more critical when complex systems are investigated. Indeed, several material features play a critical role in determining the final properties: the catalyst habit (i.e. size and shape, strictly correlated to dangling bonds of active species), surface oxygen vacancies (often correlated with *in situ* formation of active intermediates, such for instance peroxides), oxidation states of surface atoms are among the most relevant actors involved in reactant transformation during the process under investigation. It was recently highlighted^1^,^2^ that classical heterogeneous catalysis can provide suggestive concepts of surface modifications. In fact, the nanocrystalline version of well-known combinations of catalytic oxides (COX) supported onto another metal oxide (support oxide, SOX) like TiO_2_-V_2_O_5_ and TiO_2_-WO_3_, featured evident synergistic effects as concerns the enhancement of the gas-sensing response. It was argued that this occurred because, if the crystallite size of the SOX is decreased more and more, the relative electronic contribution generated by the reactions at the COX is increasingly important, due to the enhanced surface/volume atoms ratio in nanocrystalline materials. For extending this approach to surface modifications of materials, in this work we consider the SnO_2_-V_2_O_5_ system. In fact, even this system is known from heterogeneous catalysis^3^,^4^,^5^,^6^,^7^,^8^,^9^,^10^,^11^,^12^,^13^,^14^,^15^,^16^,^17^,^18^,^19^,^20^, with evidence that surface Sn-O-V bonds are the active species^6^. During the course of the study, a peculiar material structure was evidenced, featuring SnO_2_ nanocrystals wrapped by V_2_O_5_-like layers. This material architecture featured remarkably enhanced and fast adsorption and photodegradation properties with respect to naked SnO_2_, which by itself is very poorly active. Another important result was the evidence of the role of carbon residuals generated by the thermal decomposition of the organic ligands used in the synthesis step. We explicitly demonstrate, which is usually not deeply investigated in the literature, that if the residuals are not properly eliminated, the functional properties are seriously affected. Overall, this work demonstrates the proof of concept initially introduced, according to which a stable inorganic functionalization of the nanocrystal surface is able of boosting an otherwise poorer surface chemistry. This opens the way to further materials architectures suggested by heterogeneous catalysis.

## Results and Discussion

### The evolution of the materials from the as-prepared stage to the final thermal treatments

In this section the results of the characterization will be exposed, in order to build up a plausible model of the final material structure, and to show how it is developed from the as-prepared stage through the various heat-treatments, which were necessary for thermal and chemical stabilization of the materials. The criterion for the development of this section will be the increasing heat-treatment temperature and the related results. Occasionally, some results related to higher heating temperatures will be anticipated for clarity or succinctness.

**Figure 1:**
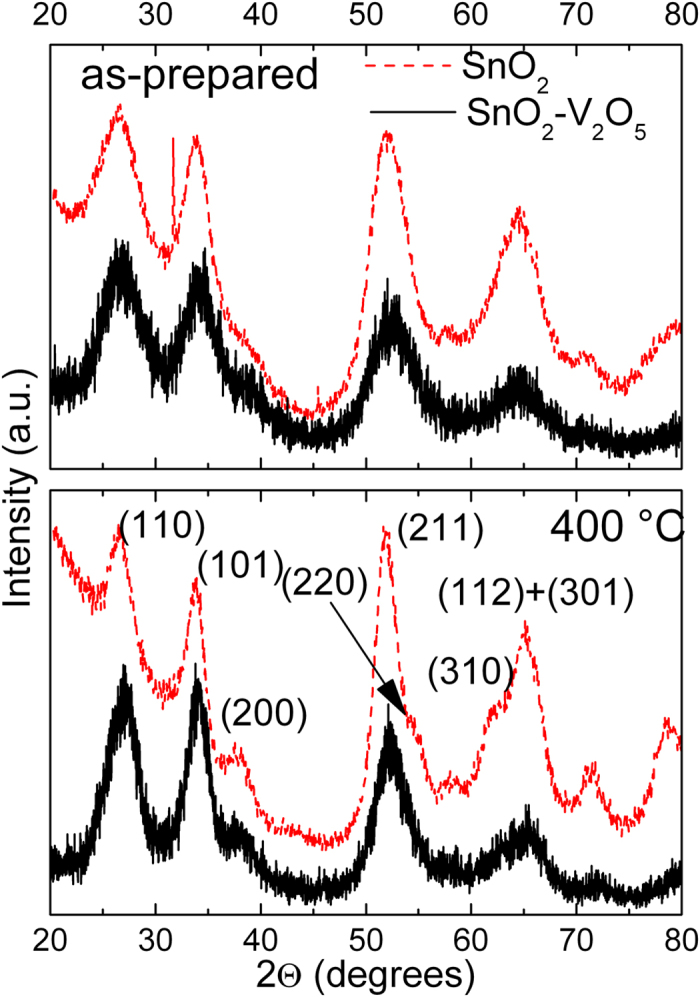
XRD patterns of the as-prepared and 400 °C SnO_2_ and SnO_2_-V_2_O_5_ samples. For clarity, not all the peaks were indexed.

The XRD patterns of the as-prepared and 400 °C SnO_2_ and SnO_2_-V_2_O_5_ samples are reported in Fig. 1. They show the reflections of only SnO_2_ in the usual cassiterite phase (JCPDS card 41–1445). No phase segregation of V_2_O_5_ or other vanadium oxides was observed, even after heating at 400 °C. Broad peaks indicate nanosized domains, and it can be noted that the pure SnO_2_ reflections become more resolved in the 400 °C sample (in particular, the (220) and (310)). Indeed, Rietveld refinement (for more details, we refer to Supplementary Figs S1 and S2 and Supplementary Tables S1 and S2) indicated that the mean SnO_2_ grain size of the pure 400 °C sample was 3.0 ± 0.5 nm, while for SnO_2_-V_2_O_5_ the size did not reach such value even after heating at 500 °C (see discussion below).

**Figure 2:**
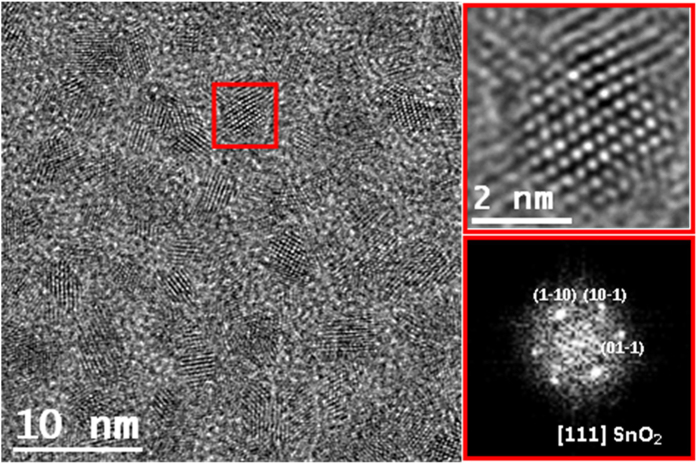
HRTEM micrograph of as-prepared SnO_2_-V_2_O_5_ sample; close-up HRTEM of a 2.7 nm single crystalline nanoparticle (red squared) and its corresponding power spectrum, revealing cassiterite SnO_2_ phase.

**Figure 3:**
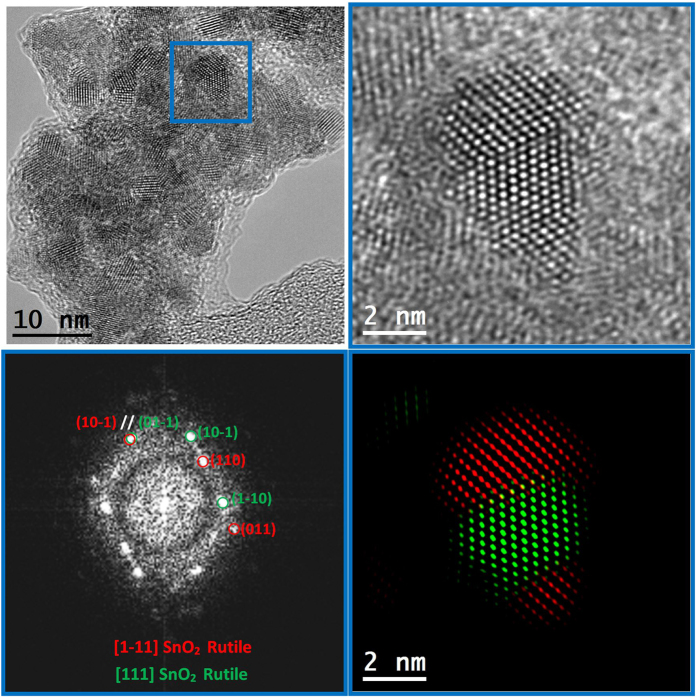
General HRTEM micrograph and detail of a twinned nanoparticle in the 400 °C SnO_2_-V_2_O_5_ sample. Its corresponding power spectrum and color phase map show two different orientations of the same cassiterite SnO_2_ phase.

Figures 2 and 3 show representative HRTEM images of the as-prepared and 400 °C SnO_2_-V_2_O_5_ samples, respectively. In the as-prepared sample, the HRTEM image contains mostly single crystalline nanoparticles, with some exceptions of bi-crystalline nanoparticles. The power spectrum analysis revealed that the nanocrystal in the squared region featured the cassiterite SnO_2_ phase (rutile structure), with lattice parameters a = b = 0.4737 nm and c = 0.3186 nm (space group = P 42/mnm). It is visualized along the [111] axis. As seen in the detailed HRTEM micrograph (it is also the case for most of the nanoparticles), the nanoparticle contains many defects, but remarkable V doping can be excluded as discussed in the following. Particle size distribution histogram, obtained by measuring about 100 nanoparticles (see Supplementary Fig. S3), showed that the nanoparticle diameter varied between 1.5 and 6 nm, with an average value of 3.5 ± 0.8 nm. Figure 3 shows a selection of HRTEM micrographs taken from the 400 °C sample.

**Figure 4:**
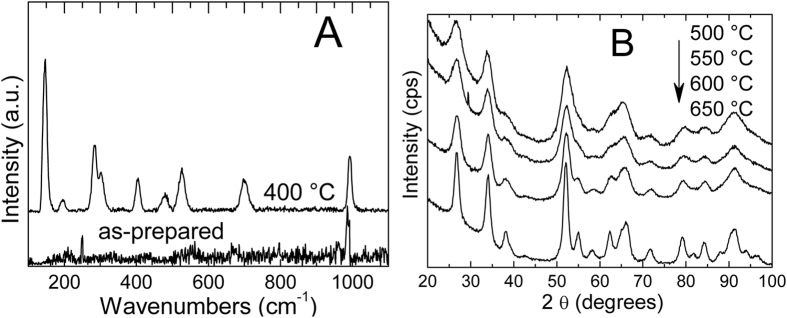
(**A**) Raman spectra measured on the as-prepared and 400 °C SnO_2_-V_2_O_5_ samples; (**B**) XRD patterns of the SnO_2_-V_2_O_5_ samples heat-treated at the indicated temperatures.

As seen in these micrographs, SnO_2_ nanocrystals, which were mostly well distributed in the as-prepared sample, were agglomerated after the applied annealing. Moreover, the agglomerates were usually covered with an amorphous layer. Average diameter of the nanocrystals was 3.98 ± 1.00 nm, only slightly larger than those of the as-prepared sample, in agreement with the XRD peak width observation. Agreement of TEM observations with the XRD conclusions confirmed that the mean size of the SnO_2_ nanocrystals in the SnO_2_-V_2_O_5_ samples underwent only a slight size increase after heat-treatment at 400 °C. More insight about the limited growth was obtained from the Raman spectra measured on the as-prepared and 400 °C SnO_2_-V_2_O_5_ samples, displayed in Fig. 4A. In the as-prepared sample, the weak and broad bands between 400 and 800 cm^−1^ can be attributed to nanocrystalline SnO_2_^21^, while a stronger peak at about 990 cm^−1^ is attributable to surface vanadyls (V = O).

It was surprising to observe the Raman curve of the 400 °C sample, which was indeed coincident with that of bulk V_2_O_5_^22^, while XRD patterns excluded that it may be present to such extent to obscure the Raman signal of SnO_2_. We concluded that the V oxide species, giving rise to the vanadyl signal in the Raman spectrum of the as-prepared sample, condensed through sol-gel reactions during the heat-treatment at 400 °C. A layer was formed onto the SnO_2_ nanocystals, as suggested by TEM observations, preventing them from further growth. Such layer is capable of giving a strong Raman signal, despite we cannot observe crystalline V_2_O_5_.

**Figure 5:**
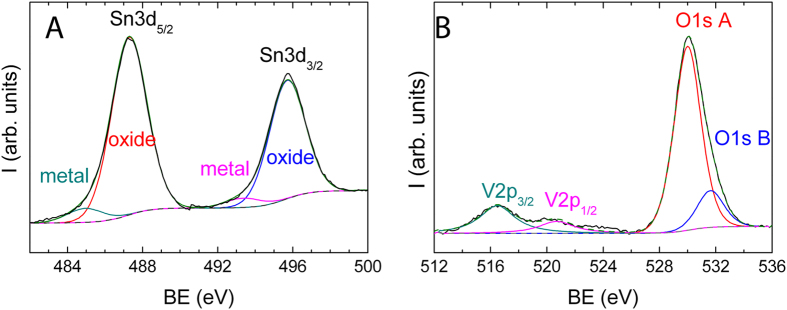
(**A**) XPS Sn3d spectrum and, (**B**) V2p and O1s spectral region of the 400 °C SnO_2_-V_2_O_5_ sample.

A possible explanation is that the local symmetry of V ions in the formed layer resembles that of V_2_O_5_, generating the specific Raman spectrum. For this reason we have been referring throughout the work to “V_2_O_5_-like” layers. For supporting this hypothesis, the SnO_2_-V_2_O_5_ samples were heat-treated at increasing temperatures, and their XRD patterns were recorded and reported in Fig. 4B. It can be seen that no additional phases could be observed up to 650 °C. More important, the cell parameters of the cassiterite structure in the SnO_2_-V_2_O_5_ samples were unchanged by the heat-treatments, as shown by Rietveld refinement (see Table 1). Since both V(IV) and V(V) ions have smaller radius than Sn(IV) in the cassiterite structure^23^,^24^, their insertion into the SnO_2_ nanocrystals would result in cell compression, but the XRD data just excluded this possibility. The analysis of the data in Fig. 4 further reinforced the hypothesis of vanadium oxide layers in the outer region of the SnO_2_ nanocrystals. The XPS spectra of Sn 3d, O1 s and V2p regions are shown in Fig. 5 for the 400 °C sample (survey spectra and fitting data for the as-prepared and 400 °C samples are reported in Supplementary Fig. S5 and Supplementary Table S3). The atomic ratio between V and Sn was almost the same before (0.19) and after annealing (0.145). In order to check the possible presence of reduced Sn^2+^ ions after annealing, the spectra of the valence band were also acquired. The shape of the valence band spectrum was not modified after annealing. Three bands could be identified in this spectrum (see Supplementary Fig. S6): V 3d and two other bands corresponding to Sn^4+^ state^25^,^26^.

Therefore, annealing only decreased the amount of oxygen present on the surface, but it did not change the chemical state of Sn, i.e. we obtained SnO_2−x_ nanocrystals with surface oxygen vacancies. The XPS analysis hence indicated that V(IV) was present from the as-prepared stage to 400 °C. Moreover, the XPS data demonstrated (see Supplementary Fig. S7) that the carbon residuals are still present after heating at 400 °C, and they can be supposed to take part to the external layers observed in the TEM images.

MAS-NMR was used for obtaining a different view of the V oxidation state. The MAS-NMR results are reported in Supplementary Fig. S8. As concerns ^51^V, in the dried sample a deshielding signal appeared around 750 ppm. It was suggested that the peak broadening can be associated to paramagnetic V(IV) centers^27^. This paramagnetic ^51^V line shift is directly proportional to the density of unpaired electrons. The paramagnetic contribution in some cases can modify the electronic environment of nuclei that are in close proximity to paramagnetic centers^28^. This was further confirmed by the remarkably broadened ^119^Sn signal of the as-prepared sample. For this reason we hypothesized Sn-O-V bonds in the as-prepared sample, with V in the paramagnetic V(IV) state. After the heat-treatment at 400 °C, a ^119^Sn peak at −605 ppm that corresponds to bulk SnO_2_ was found^29^. As concerns the ^51^V signal, after the heat-treatment known signals corresponding to V(V) placed in octahedral sites (V_2_O_5_, −620 ppm) and in tetrahedral sites (VO_4_^−3^, −585 ppm) were found^30^. We will comment below about this discrepancy with the XPS data.

The study of the materials evolution was completed by the XPS studies of the 500 °C samples. The V2p, O1s and valence band spectra are shown in Supplementary Fig. S9. Elemental composition data, reported in Table 2, confirmed the remarkable carbon decrease after heating at 500 °C, which was then selected as the proper temperature for materials used in functional tests. More important, the V oxidation state can be entirely identified as V(V), as confirmed by its BE value and the distance between the V2p_3/2_ and V2p_1/2_ peaks, i.e. ∆ = 7.4 eV^31^. As concerns tin, it was in + 4 state: this is confirmed by its BE value and the valence band shape which corresponds perfectly to the one of SnO_2_^32^. The O/Sn ratio rose up again to about 2, indicating re-oxidation of the sample after the previously observed oxygen deficient situation at 400 °C. The fact that after heating at 500 °C the carbon concentration was very low while the SnO_2_ grains increased only slightly, definitely reinforced the hypothesis of inorganic surface coating by V_2_O_5_ layers, suggested by the Raman analyses in Fig. 4. Concerning this point, we note that the Raman spectra of the samples heat-treated up to 650 °C still display a structure resembling that of V_2_O_5_, despite the distorted shape and an unidentified signal at about 860 cm^−1^ (see Supplementary Fig. S11), while XRD always excluded this phase (Fig. 4). Our hypothesis about the presence of external V_2_O_5_-like layers after heating at 500 °C is also supported by the observation that, if V(V) ions were only incorporated into the cassiterite structure, the corresponding equations, in Kröger-Vink notation, would be:


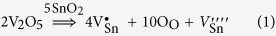



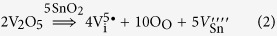


for substitutional (eq. 1) and interstitial (eq. 2) vanadium incorporation, respectively. Here V_Sn_, V_i_, and O_O_ indicate substitutional and interstitial vanadium and regular oxygen sites, respectively, while the final italicized symbols indicate tin vacancies. The consequent formation of Sn vacancies, to such a large extent as required by the observed high V/Sn relative concentration (Table 2), is not compatible with the observed XPS stoichiometry. Moreover, from a structural point of view, the formation of numerous vacancies in the small SnO_2_ nanocrystals (2.8 nm after heating at 500 °C) would be very unfavorable. On the other hand, the atomic ratio V/Sn obtained from the XPS data is about 20% (Table 2). If we consider that until now there was no evidence of extensive V doping of tin oxide, such a high concentration of vanadium further points to the external layers of vanadium pentoxide. Segregated, amorphous V_2_O_5_ is also to be excluded since it should crystallize at much lower temperatures^33^.

**Figure 6:**
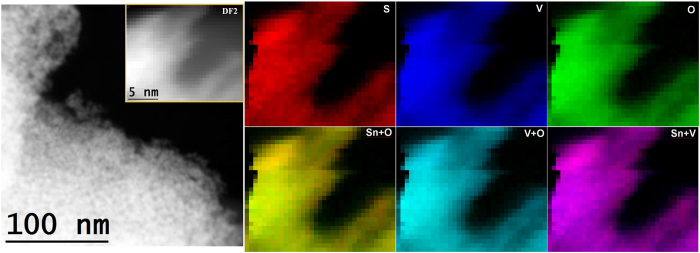
EELS chemical composition maps obtained from the yellow rectangled area of the STEM-DF2 (the inset of left image) micrograph. Individual Ti (red), V (blue) and O (green) maps and their composite.

For further clarifying the structural and chemical situation of the 500 °C SnO_2_-V_2_O_5_ sample, TEM/EELS investigations were carried out. First of all, the images confirmed that the nanocrystals did not experience dramatic growth, with a mean size in agreement with the XRD observations. Second, the Sn, V and O elements signals were distributed evenly in the investigated regions, as shown in Fig. 6 (more results are reported in Supplementary Fig. S13). The STEM-DF2 image shows the nature of the nanoparticles and the EELS chemical composition maps obtained from the yellow squared area of the micrograph. It is clear that the Sn, V and O elements signal are distributed evenly in the nanoparticles area. Coupled with the lack of V_2_O_5_ phase segregation, this result was interpreted as an evidence of uniform vanadium coverage of the SnO_2_ nanocrystals. Very interestingly, some SnO_2_ nanocrystals were found to be in the orthorhombic Pbcn crystallographic phase, which is observed usually in high pressure experiments or in very small structures^34^. It is not surprising that no XRD signal was detected, since the peaks of this phase are largely overlapped with the broad cassiterite signals. This unusual phase transition may be interpreted as further demonstration of the influence of the surface modification by vanadium oxide species: the surface contribution to the Gibbs free energy of the system is very important for small sized structures, and may result in important modifications of the polymorph stability^35^. With the help of the data collected until now, together with FTIR and thermal analyses data shown in Supplementary Fig. S14, we are ready to propose a phenomenological view of the material formation pathways.

When the as-prepared SnO_2_ nanocrystals are reacted in the solvothermal step with the V precursor solution, surface Sn-O-V bonds are formed (Raman, MAS-NMR) by cross-linking of surface Sn-OH with V-OH groups. These species are isolated (Raman and FTIR signals of only vanadyls) and comprise only V(IV) ions (XPS, MAS-NMR). During the heat-treatment up to 400 °C, the oleic acid ligands are removed to large extent and/or decomposed (FTIR, thermal analyses and XPS), but the more naked surfaces do not favor SnO_2_ sintering and/or appreciable V migration into the cassiterite structure, not even at high temperatures (XRD data of Fig. 4). Instead, the V precursor prefers to self-condense over the SnO_2_ surface (Raman data in Fig. 4), wrapping around the SnO_2_ nanocrystals and preventing them from further sintering (HRTEM results). Raman and MAS-NMR data indicate the presence of V(V) species in the V_2_O_5_ coordination, but XPS data point to a more complex situation, where mixed valence V species are most probably present. Concerning this point, it is interesting to observe the oxygen defective structure of SnO_2_ revealed by XPS after the heat-treatment at 400 °C. Oxygen vacancies may stabilize the surface vanadium species toward lower oxidation states: electron density is more efficiently retained on the vanadium centers in absence of electronegative oxygen neighbors. This would point to higher concentration of V(IV) species near the nanocrystal surface, where the oxygen defects are present, and largely influencing the XPS signal. Instead, after heating at 500 °C, only V(V) was observed in XPS spectra while carbon concentration was dramatically lowered. This result reinforced the hypothesis of carbon residuals stabilizing lower vanadium oxidation states at lower heating temperatures.

After heating at 500 °C, the final material was obtained, described as follows: stoichiometric SnO_2_ nanocrystals, with minimized carbon content, wrapped in V_2_O_5_ layers, without any V(IV) component and extremely hindered sintering.

### Photodegradation properties

**Figure 7:**
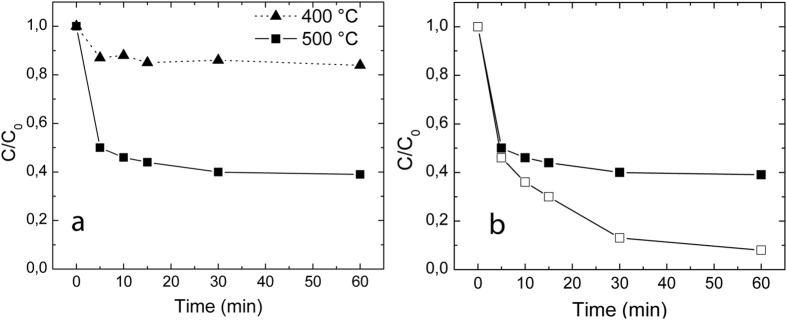
(**a**) Photodegradation of RhB 10^−5^ M under simulated solar light by 400 °C (triangles) and 500 °C (squares) SnO_2_-V_2_O_5_ samples; (**b**) photodegradation of RhB 10^−5^ M (full markers) and 10^−7^ M (empty markers) by 500 °C SnO_2_-V_2_O_5_ sample.

SnO_2_-V_2_O_5_ samples heat-treated at 400 °C and 500 °C were investigated as potential photocatalysts towards the photodegradation of rhodamine B in water, used as model system for water pollutants removal. Obvious differences in photocatalytic activity between the two samples are visible by observing the photodegradation curves reported in Fig. 7a (RhB concentration as high as 10^−5^ M). The 500 °C sample showed a nice capability of degradating the dye (about 60% of RhB was converted in less than 1 h under simulated solar light irradiation). On the contrary, 400 °C sample showed poor catalytic activity and even after 2 h of reaction more than 80% of the initial RhB amount was still identified by spectrophotometric analysis. Moreover, reaction course sustained by 400 °C sample displayed anomalous behavior, showing small fluctuations in the values of amount of converted RhB. Considering the previous discussion on materials, the reduced capability of the 400 °C sample to convert RhB is reasonably ascribed to the carbon residuals present on the surface. Presence of carbon residual in high amount on catalyst surface not only possibly lowers the number of catalytically active sites available for dye adsorption, but can also impair photogenerated charge exchange between the two partners and between the catalyst and the potentially active species in water.

**Figure 8:**
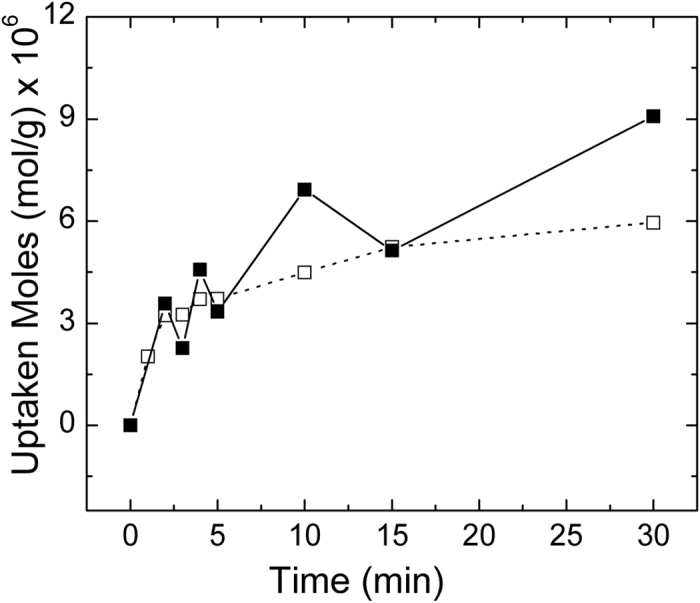
Dye uptake curve of 500 °C sample under dark (full markers: RhB 10^−5^ M; empty markers: 10^−7^ M).

Previous literature on the topic has indeed pointed out as a possible mechanism for RhB photodegradation the formation of oxidative radicals from water (such as OH^•^ and HO_2_^•^) by oxidation through holes generated under semiconductor exposure to light of proper energy (higher than the corresponding band gap) and the reduction of O_2_ dissolved in water by the transfer of photo-induced electrons on the catalyst surface^36^. High adsorption of RhB on material surface is then relevant since proximity can promote both a rapid redox exchange between the active species generated in water close to catalyst surface and direct oxidation of dye by the catalytically active materials. The 500 °C sample was further investigated by decreasing the initial RhB concentration by two orders of magnitude (down to 10^−7^ M): under these conditions (Fig. 7b) SnO_2_-V_2_O_5_ was able to completely degrade the dye in about 1 h. Dye uptake capability of 500 °C sample was moreover explored for the two RhB concentrations under investigation (Fig. 8). RhB uptake course was found to follow the typical trend identified for dye loading on metal oxides observed in previous studies^37^,^38^ following a pseudo-first order kinetics. Dye uptake presents a regular trend when the RhB concentration is kept at 10^−7^ M, while fluctuations are identified at higher dye concentration, suggesting that in these conditions more than one monolayer is present on catalyst surface, possibly to due RhB aggregation phenomena, which are however subjected to de-aggregation under vigorous stirring.

**Figure 9:**
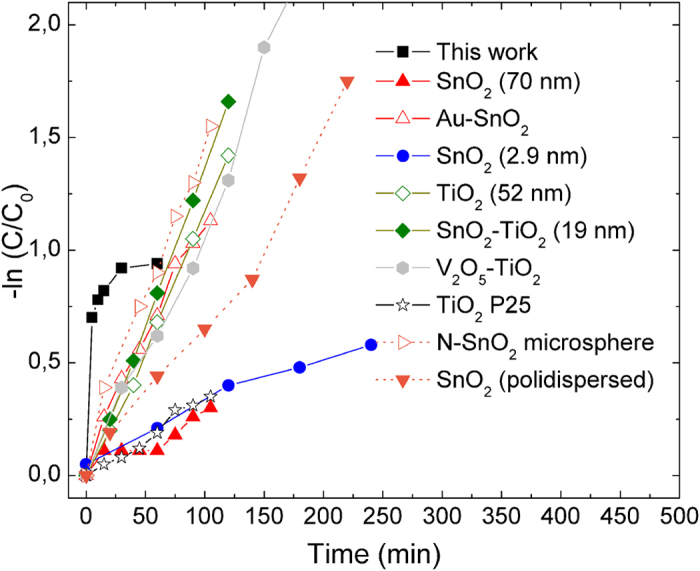
Comparison of photocatalytic activity of 500 °C sample (black squares) with literature data: red and blue markers: digitized data from Wu *et al*.^36^; green markers: digitized data from Messih *et al.*^41^; grey markers: digitized data from Dai *et al.*^42^; orange markers: digitized data from Li *et al.*^43^. Markers are experimental data, lines are guide for the eye.

Adsorption of RhB can also be used to estimate the sample surface area, considering a molecular area as high as 1.6 nm^2^ for RhB^39^: evaluation of surface area of 400 °C and 500 °C sample by this approach gave differences of one order of magnitude (about 8.5 m^2^/g and 64 m^2^/g, respectively, the latter value in very good agreement with the 70.3 m^2^/g value obtained by BET), which is again attributed to the carbon residual identified on the surface of the sample treated at lower temperature, thus indicating this feature as extremely critical in determining the chemical nature of the nanocomposite systems as well as their functional performances. Both single SnO_2_ and V_2_O_5_ have been investigated as photocatalysts, but, in general, direct comparison of photocatalytic data may be difficult due to different experimental conditions adopted by different Authors. However, we tried to compare the functional results obtained in the present study with similar investigations conducted with SnO_2_, V_2_O_5_ and SnO_2_-modified nanostructures applied to photodegradation of RhB 10^−5^ M (comparison of photocatalytic data is reported in Fig. 9). TiO_2_ is usually considered as a benchmark material in photocatalysis^40^ and TiO_2_ P25 is often used as a reference material, showing however a rather slow kinetics in RhB degradation under UV irradiation (empty stars in Fig. 9). TiO_2_ nanoparticles of uniform size as high as 52 nm prepared via sol-gel have been also investigated^41^, showing a nice photocatalytic activity (empty rhombs in Fig. 9), further improved under UV irradiation when mixed SnO_2_-TiO_2_ nano-oxides were similarly synthesized (full rhombs in Fig. 9).

Improvement was attributed to reduced crystallite size induced by tin (down to 19 nm) and enhancement of surface properties of the composed materials. Pure SnO_2_ has been as well exploited, by either investigating commercial particles (size of 70 nm or polydispersed) or specifically designed nanosystems (size of 2.9 nm). Comparison of literature data suggests that decrease in system sizes does not heavily affect the photocatalytic performances of SnO_2_ (red triangles and blue circles in Fig. 9), while polydispersion (size from 20 to about 300 nm) provides for more efficient system (inverted orange triangles in Fig. 9)^43^. Remarkable improvements in photocatalytic activity of SnO_2_ have instead been obtained by fabrication of Au-SnO_2_ nanostructures (overall sizes of about 57 nm, empty red triangles in Fig. 9)^36^ and by nitrogen doping of SnO_2_ hollow microspheres (empty orange triangles in Fig. 9)^43^. In both cases, enhancement of photocatalytic performances was attributed to enhanced visible light absorption, induced by either plasmonic effect or doping. Very few studies report about the application of V_2_O_5_ to RhB photodegradation. Jang *et al*.^44^ investigated the RhB adsorption and photocatalytic degradation on different crystalline V_2_O_5_ forms, observing rather good adsorption capability (21 to 61% of the initial RhB amount in solution, according to the type of material under investigation, i.e. shape and surface area) but moderate photocatalytic activity (maximum 30% after 1 h of visible light irradiation with a maximum at λ = 380 nm). Wang *et al*.^45^ investigated 1D TiO_2_-V_2_O_5_ branched nanostructures, showing good catalytic activity under visible light irradiation (90% RhB was photodegraded within 180 min), attributed to both increased visible light absorption (compared with bare 1D TiO_2_ structures) induced by V_2_O_5_ and enhanced exciton separation in the composite material (grey hexagons in Fig. 9). The 500 °C sample showed an impressive speed of RhB conversion in the first 30 minutes of reaction, much higher than the bare SnO_2_, doped and complex catalysts reported for comparison, reaching then a plateau. Analysis of reaction course clearly indicated that more than one process was occurring in the present case, since the reaction is not following pseudo-first order kinetics, as usually reported for semiconducting metal oxides. During the reaction course we moreover remarked a hypsochromic shift of the absorption maximum over the time (see Fig. S15 in the supplementary information). Analogous shifts have been previously reported by several investigators^46^ and attributed to species formed in the reaction mixture under the action of the catalyst. In particular, the analysis of the hypsochromic shift observed in the present study (from 554 nm to 540 nm after 60 min reaction, being the latter attributable to rhodamine) strongly suggest a N-deethylation path of the adsorbate species for the photodegradation of RhB under the studied conditions, through radical species formed via electron transfer from the excited dye in its singlet state to the conduction band of the catalyst.

**Figure 10:**
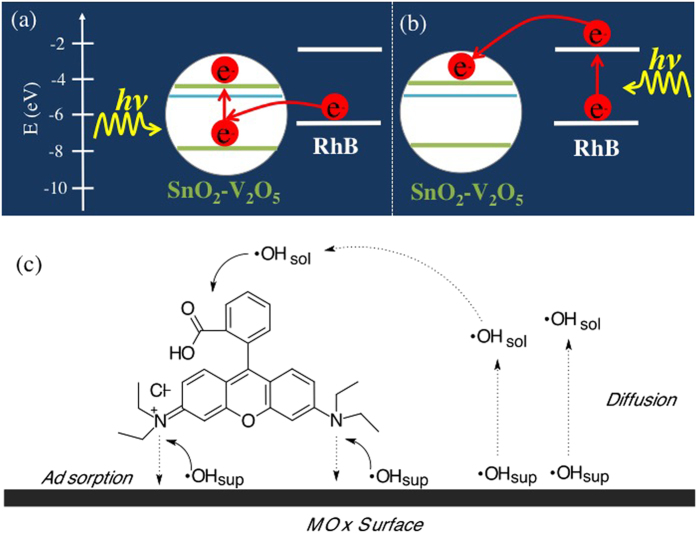
(**a**) and (**b**) Energy alignment scheme for the valence and conduction bands of SnO_2_-V_2_O_5_ (energy gap of pure SnO_2_ is considered together with a intra band-gap “level” representing the defects induced by V_2_O_5_ surface layers, light blue line) and HOMO and LUMO of RhB, showing the processes occurring under irradiation of (**a**) metal oxide and (**b**) RhB. (**c**) Subsequent formation and reaction of •OH radicals.

In this respect, it is worth noting the under simulated solar light irradiation both the dye and the catalyst can get excited (see Fig. 10a,b). When RhB is in its excitation configuration an electron can be injected from the RhB LUMO (−2.73 eV) into the MO_X_ CB; on the other hand, excitation of the metal oxide leaves a hole in its VB that can be compensated by an electron from the HOMO (−4.97 eV) of the dye. This possibility has been previously verified for another semiconductor (CdS)^47^ and the main requirement is a favorable energy alignment, which is the present case. However, in case of metal oxide excitation, the photogenerated electron-hole pair is subjected to fast recombination within the metal oxide significantly diminishing, the formation of •OH radicals. In case of dye excitation, one electron can be injected into the metal oxide conduction band and readily captured by the oxygen present in the solution thus producing OH radicals on the surface (•OH_surf_), which can easily attack the surface adsorbed RhB molecules and promoting the N-deethylation. Under prolonged continuous irradiation, the excess of •OH radicals on the MO_X_ surface diffuses in the solution (•OH_sol_) and participates in the degradation of RhB.

On this basis, it is now possible to introduce some more detailed hypothesis about the overall influence of V_2_O_5_ surface modification and any synergy with the SnO_2_ nanocrystals. The SnO_2_ nanocrystals are surface modified by V_2_O_5_ layers, which do not possess the extended bulk crystalline arrangement, but only a local V_2_O_5_-like geometry, as illustrated in the interpretation of the characterization data. This suggests that any light absorption modification in the visible by V_2_O_5_ cannot be interpreted on the bulk bandgap value for V_2_O_5_. It is clear that such modification enhances the photocatalytic activity, by just increasing the formation rate of charges available for the above suggested mechanism. On the other hand, these layers do not exist as independent chemical species, and they need the support of the SnO_2_ nanocrystals. In this sense, the photocatalytic activity emerges as “synergistic effect” due to the cooperation of the two components. The SnO_2_ nanocrystals, of course, do not act simply as spatial support, but influence the electron density on the V cations, which further justifies the hypothesis of synergistic effect. It is not possible to go beyond this phenomenological description, without a precise calculation of the energy levels introduced by the surface V ions in the overall structure. Nevertheless, we can still represent them as done in Fig. 10 by a blue line. It is readily clear that these states may strongly influence the photocatalytic performance. In particular, our hypothesis is that V_2_O_5_ acts as a source of surface trap states, which can slow down the photogenerated charge recombination at SnO_2_, previously discussed in the discussion of Fig. 10, thus supporting the generation of OH radicals in the reaction mixture.

## Conclusions

By using hydrolytic sol-gel chemistry in solvothermal conditions, it was possible to link covalently the V_2_O_5_-like species to the surface of SnO_2_ nanocrystals. The collected experimental data pointed to a peculiar structure after heating at 500 °C, with SnO_2_ nanocrystals embedded into V_2_O_5_-like layers. This structure generated a synergistic enhancement of the surface properties of the material. Photocatalytic degradation of rhodamine B showed impressive capability of the 500 °C sample to convert the dye at low concentration and good skill as photocatalyst for higher RhB amounts. Very good adsorption was moreover observed under dark conditions: this is also a relevant property, since the composite could in principle be used to remove dye pollutants by adsorption with no need of irradiation.

## Experimental

SnO_2_ nanocrystals were synthesized by modifying a previously described sol-gel precipitation in dodecylamine^48^. Briefly, 2 mL of tin chloromethoxide solution were dropped into 10 mL of n-dodecylamine at room temperature. The white precipitate was extracted by methanol, washed 2 times in acetone, and then dispersed into 10 mL of oleic acid (technical grade). Then, 0.5 ml of vanadium chloromethoxide, prepared as previously described^33^, was added. The resulting suspension was poured into a glass vial and inserted into an autoclave, kept for 2 h at 250 °C. After cooling, the black product was extracted by methanol, washed with acetone and dried in air at 90 °C. Eventually, the product was heat-treated for 1 h in air at various temperatures in a muffle furnace, in a porcelain crucible. Pure SnO_2_ materials were prepared in analogous way, by skipping the addition of the V precursor.

XRD data were collected in Debye-Scherrer geometry on a Rigaku RINT 2500 diffractometer, equipped with an asymmetric Johansson monochromator (Ge 111 reflection) for Cu Kα_1_ radiation (λ = 1.54056 Å) and a D/tex Ultra detector. The rotating anode source was operated at 50 kV, 200 mA. The powder sample was introduced in a 0.3 mm diameter Lindemann glass capillary, set to rotation during data collection. The whole XRD profiles were fitted by the FullProf software [ https://www.ill.eu/sites/fullprof/], using a Rietveld approach taking into account the instrumental resolution function (IRF, i.e. the instrumental broadening). A LaB_6_ powder sample from NIST was used as a standard to evaluate the IRF.

Raman spectroscopy was performed by means of a Jasco NRS-5100 spectrometer with a green laser in a micro-Raman configuration with 100x objective and with a laser power of 10 mW. High resolution transmission electron microscopy (HRTEM) analyses of the powders were carried out by a field emission gun FEI Tecnai F20 microscope, working at 200 kV and with a point-to-point resolution of 0.19 nm. Large area XPS measurements at 20 eV pass energy were performed in an Escalab MkII spectrometer (VG Scientific Ltd., UK) equipped with a 5-channeltron detection system. The samples were pressed on the grated Au foil (99.99%) fixed on the standard Escalab holder stubs. An unmonochromatized Al Kα radiation source (1486.6 eV) was used for the sample excitation. The binding energy (BE) scale was calibrated by measuring the reference peaks of Au 4f_7/2_ (84.0 ± 0.1 eV) from the supporting foil. The spectroscopic data were processed by Avantage v.5 software (Thermo Fisher Scientific, UK).

^51^V and ^119^Sn NMR spectra were recorded in a 4 mm zirconia rotors at room temperature by a wide-bore Varian Infinity Plus 400 (9.4 T) spectrometer, operating at 105.152 and 148.98 MHz respectively. A single pulse was the sequence used in all the cases. The p/2 pulse was 4 μ for ^51^V and 2 μ for ^119^Sn, spinning at 12 and 7 KHz respectively. A recycling delay of 1 s was used in ^51^V NMR spectra with 20.000 transients; for ^119^Sn, a delay of 10 s and 300.000 accumulations were used, for allowing Signal/Noise ratios better than 20. For ^51^V, the chemical shift was determined with ammonic metavanadate as external reference (−571.5 ppm) and VOCl_3_ as internal reference (0 ppm). ^119^Sn NMR spectra were referenced to SnMe_4_ (0 ppm).

Thermal analyses were carried out by a SDT Q-600 thermal balance from TA instruments under air flow of 100 mL/min and thermal ramp of 10 °C/min.

Fourier Transform Infrared (FTIR) measurements were carried out by a Nicolet 6700 spectrometer in diffuse reflectance setup, after dispersing the sample powders in KBr.

Photocatalytic activity of prepared materials was evaluated by the photodegradation of rhodamine B (RhB) in water (investigated concentrations: 10^−5^ and 10^−7^ M) under simulated solar light irradiation. An ABET 2000 solar simulator at AM 1.5 G (100 mW cm^−2^) calibrated with a silicon reference cell was used as solar light source. Before irradiation, 50 mg of the active materials were dispersed in 100 ml of RhB solution and stirred vigorously under dark, in order to reach the adsorption/desorption equilibrium. Reaction mixture was then irradiated for 120 min and aliquots were collected at given time intervals and analyzed for quantification of residual RhB by means of a PG80 spectrophotometer. Quantification of dye degradation was made using a calibration curve considering six standard solutions at different concentration analyzed in triplicate.

## Additional Information

**How to cite this article:** Epifani, M. *et al*. Inorganic Photocatalytic Enhancement: Activated RhB Photodegradation by Surface Modification of SnO_2_ Nanocrystals with V_2_O_5_-like species. *Sci. Rep.* **7**, 44763; doi: 10.1038/srep44763 (2017).

**Publisher's note:** Springer Nature remains neutral with regard to jurisdictional claims in published maps and institutional affiliations.

## Supplementary Material

Supplementary Information

## Figures and Tables

**Table 1:** Mean SnO_2_ grain size and cassiterite cell parameters resulting from the Rietveld refinement of the XRD patterns in Fig. 4B.

Temperature	Mean SnO_2_ grain size (nm)	Cell parameters (±0.005)
500 °C	2.8 (pure SnO_2_: 5.28)	a = 4.712 = b c = 3.171
550 °C	3.5	a = 4.712 = b c = 3.169
600 °C	4.0	a = 4.709 = b c = 3.173
650 °C	6.1	a = 4.711 = b c = 3.172

**Table 2:** Summary of XPS analysis of the 500 °C SnO_2_-V_2_O_5_ sample.

Peak	BE, eV	FWHM, eV	Atomic, %	Bond
C1s	284.5	3.1	6.4	graphitic
O1s A	530.6	1.6	44.0	oxides
O1s B	532.0	1.6	11.1	OH^−^
Sn3d_5/2_ A	486.7	1.4	27.5	SnO_2_
V2p_3/2_ A	517.4	1.9	5.6	V_2_O_5_
