# Corrigendum: Inorganic Photocatalytic Enhancement: Activated RhB Photodegradation by Surface Modification of SnO_2_ Nanocrystals with V_2_O_5_-like species

**DOI:** 10.1038/srep46855

**Published:** 2017-06-15

**Authors:** Mauro Epifani, Saulius Kaciulis, Alessio Mezzi, Davide Altamura, Cinzia Giannini, Raül Díaz, Carmen Force, Aziz Genç, Jordi Arbiol, Pietro Siciliano, Elisabetta Comini, Isabella Concina

Scientific Reports 7: Article number: 44763; 10.1038/srep44763 published online: 03 16 2017; updated: 06 15 2017.

In this Article, an affiliation for Jordi Arbiol was omitted. The correct affiliations for Jordi Arbiol are listed below:

Catalan Institute of Nanoscience and Nanotechnology (ICN2), CSIC and The Barcelona Institute of Science and Technology (BIST), Campus UAB, Bellaterra, 08193 Barcelona, Catalonia, Spain.

ICREA, Pg. Lluís Companys 23, 08010 Barcelona, Catalonia, Spain.

