# Supplementary material for: Inorganic Photocatalytic Enhancement: Activated RhB Photodegradation by Surface Modification of SnO2 Nanocrystals with V2O5-like species
Source: Sci Rep. 2017 Mar 16;7:44763. doi: 10.1038/srep44763 (PMC5353695; doi:10.1038/srep44763)
Supplement: Supplementary Information [file srep44763-s1.pdf]

Inorganic Photocatalytic Enhancement: Activated RhB Photodegradation by Surface Modification of SnO_2 Nanocrystals with V_2O_5-like species

Mauro Epifani[1,a], *Saulius Kaciulis*[2], *Alessio Mezzi*[2], *Davide Altamura*[3], *Cinzia Giannini*[3], *Raül Díaz*[4], *Carmen Force*[5], *Aziz Genç*[6], *Jordi Arbiol*[6,7], *Pietro Siciliano*[1], *Elisabetta Comini*[8,9], *Isabella Concina*[8,9,10]

[1]Istituto per la Microelettronica e i Microsistemi, IMM-CNR, Via Monteroni, 73100 Lecce, Italy; [a]*mauro.epifani@le.imm.cnr.it*

[2]Istituto per lo Studio dei Materiali Nanostrutturati, ISMN–CNR, PO Box 10, 00015 Monterotondo Stazione, Roma, Italy

[3]Istituto di Cristallografia, IC-CNR, Via Giovanni Amendola, 122/O, 70126 Bari, Italy

[4]Electrochemical Processes Unit, IMDEA Energy Institute, Avda. Ramón de la Sagra, 3 28935 Móstoles, Spain

[5]NMR Unit, Centro de Apoyo Tecnológico, Universidad Rey Juan Carlos, c/Tulipán, s/n, 28933 Móstoles, Spain

[6]Institut de Ciència de Materials de Barcelona, ICMAB-CSIC, Campus de la UAB, 08193 Bellaterra, Spain

[7]Institució Catalana de Recerca i Estudis Avançats (ICREA), Passeig Lluís Companys, 23, 08010 Barcelona, CAT, Spain

[8]Department of Information Engineering, Brescia University, Via Valotti 9, 25133 Brescia, Italy

[9]CNR-INO SENSOR Lab, Via Branze 45, 25123 Brescia, Italy

[10] Luleå University of Technology, 971 98 Luleå, Sweden

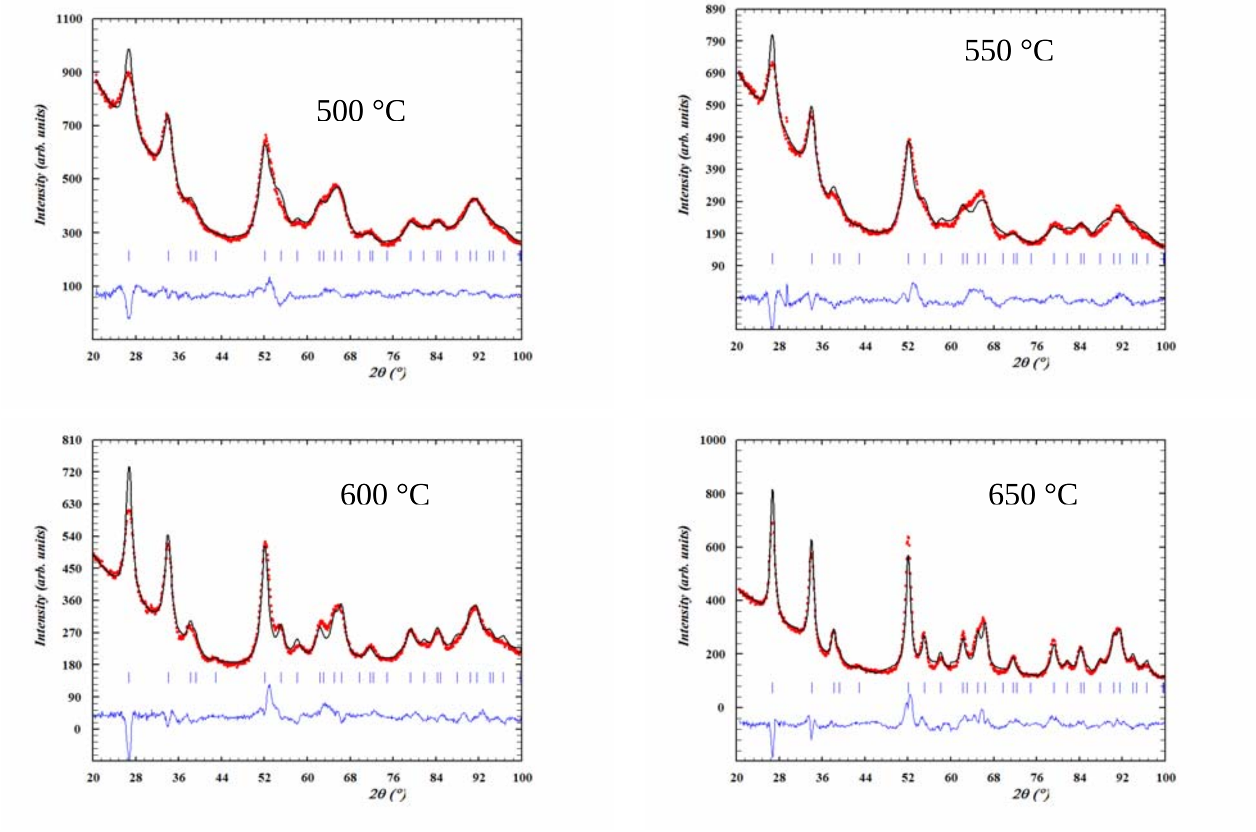

Figure S1: Rietveld refinement of the XRD patterns measured on the SnO_2-V_2O_5 samples heat-treated at the indicated temperatures.

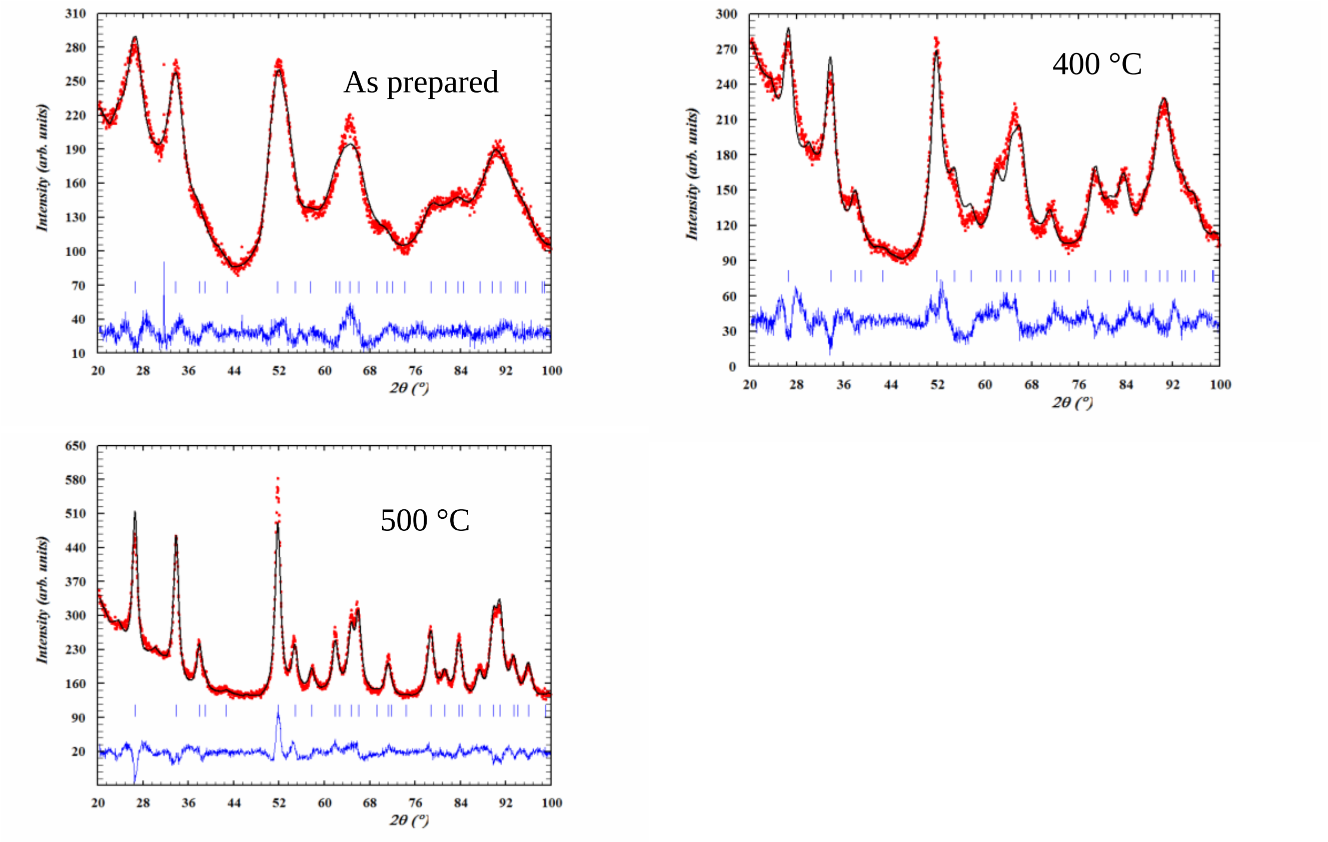

Figure S2: Rietveld refinement of the XRD patterns measured on the pure SnO_2 samples heat-treated at the indicated temperatures

Table S1: results of the Rietveld refinement of the patterns shown in **Figure S1**.

**SAMPLE Heating temperature**	**Unit cell**		**Average Apparent size (Å)/ Anisotropy (Å)**		**%**	
	SnO_2 cassiterite	V_2O_5	SnO_2 cassiterite	V_2O_5	SnO_2 cassiterite	V_2O_5
500 °C	a=4.712442=b<br>c=3.171019<br>α=90.000000<br>β=90.000000<br>γ=90.000000	-	28.12	-	100	-
550 °C	a=4.711814=b<br>c=3.169439<br>α=90.000000<br>β=90.000000<br>γ=90.000000		35.49			
600 °C	a=4.708707=b<br>c=3.172845<br>α=90.000000<br>β=90.000000<br>γ=90.000000		40.47			
650 °C	a=4.711174=b<br>c=3.172478<br>α=90.000000<br>β=90.000000<br>γ=90.000000		61.45			

Table S2: results of the Rietveld refinement of the patterns shown in **Figure S2.**

**SAMPLE**	**Unit cell**		**Average Apparent size (Å)/ Anisotropy (Å)**		**%**	
	SnO_2 cassiterite	V_2O_5	SnO_2 cassiterite	V_2O_5	SnO_2 cassiterite	V_2O_5
As-prepared	a=4.7267=b<br>c=3.2034<br>α=90.000000<br>β=90.000000<br>γ=90.000000	-	17.43 / -	-	100	-
400 °C	a=4.7293=b<br>c=3.1960<br>α=90.000000<br>β=90.000000<br>γ=90.000000		30.21 / -		100	
500 °C	a=4.7333=b<br>c=3.1877<br>α=90.000000<br>β=90.000000<br>γ=90.000000		52.80 / -		100	

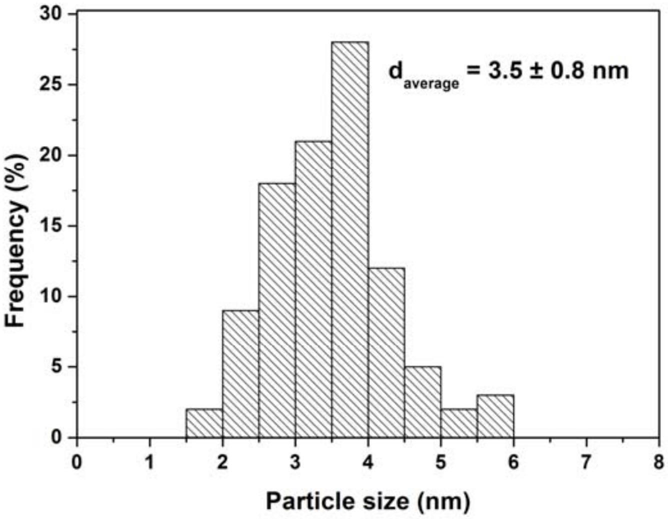

Figure S3: SnO_2 size distribution in the dried SnO_2-V_2O_5 sample obtained by measuring 100 nanoparticles in the related TEM micrographs.

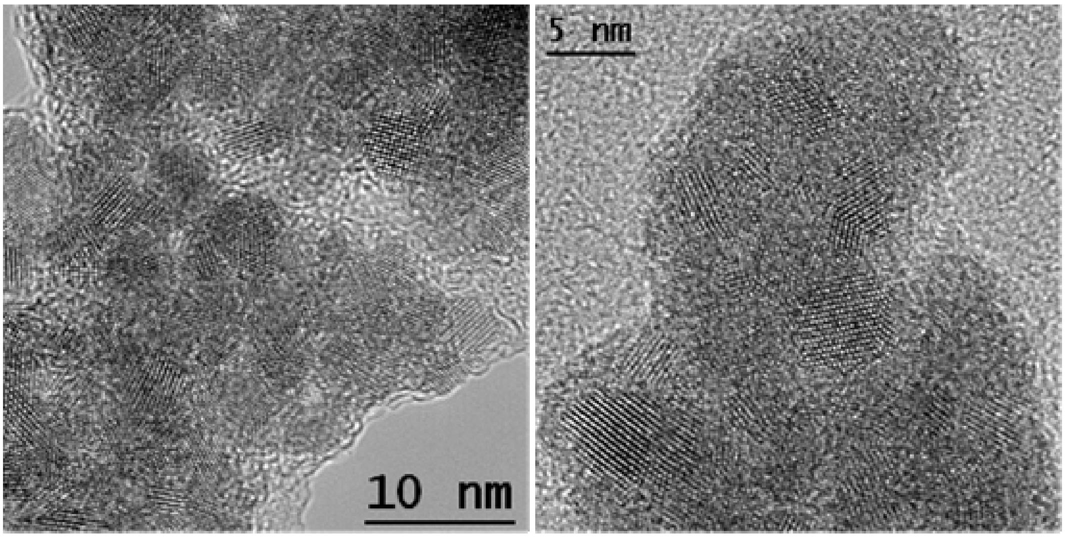

Figure S4: general view TEM micrographs of the SnO_2-V_2O_5 sample heat-treated at 400 °C, where the enwrapping of the SnO_2 nanocrystals is shown.

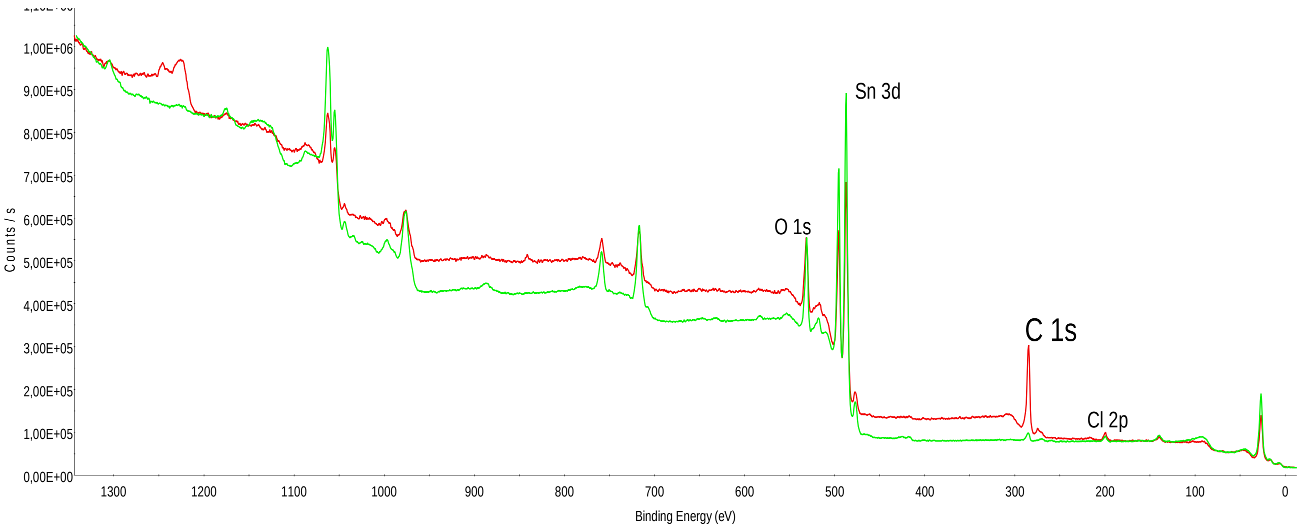

Figure S5: Survey XPS spectra measured onto the dried (red) and 400 °C (green) SnO_2-V_2O_5 samples.

Table S3: summary of XPS analysis of the as-prepared and 400 °C SnO_2-V_2O_5 samples

	**As prepared**			**400 °C**		
**Name**	Peak	At.%	Bond	Peak	At.%	Bond
**$Sn3d_{5/2}$ A**	487.3	10.5	Sn(IV) oxide	486.4	26.8	Sn(IV) oxide
**$Sn3d_{5/2}$ B**	484.7	0.9	metal	484.0	1.6	metal
**O1s A**	531.6	22.6		530.0	35.6	Oxide
**O1s B**				531.8	6.6	OH groups
**$V2p_{3/2}$ A**	516.7	2.0	V(IV) oxide	516.3	3.9	V(IV) oxide
**C1s A**	285.0	60.1	aliphatic C-C, C-H	285.0	20.5	aliphatic C-C, C-H
**C1s B**	289.2	1.1	carboxyl, carbonate	288.0	2.1	carboxyl
**Cl2p3**	199.2	2.8		198.9	2.9	

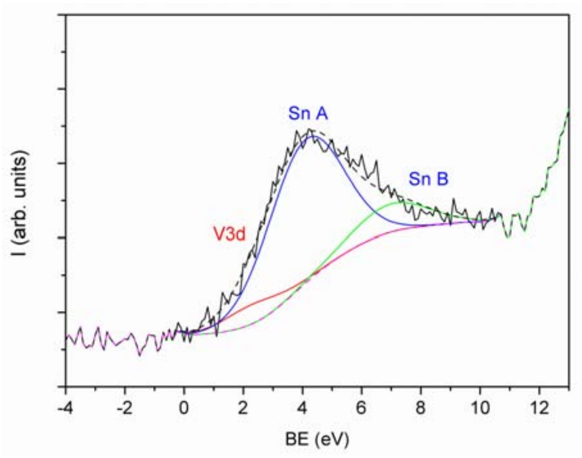

Figure S6: Sn valence band spectrum of the 400 °C SnO_2-V_2O_5 sample.

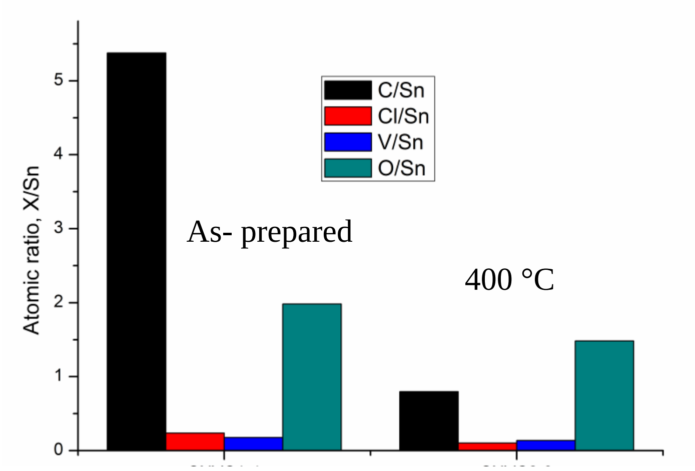

Figure S7: relative concentrations of the main elements in the as-prepared and 400 °C SnO_2-V_2O_5 samples.

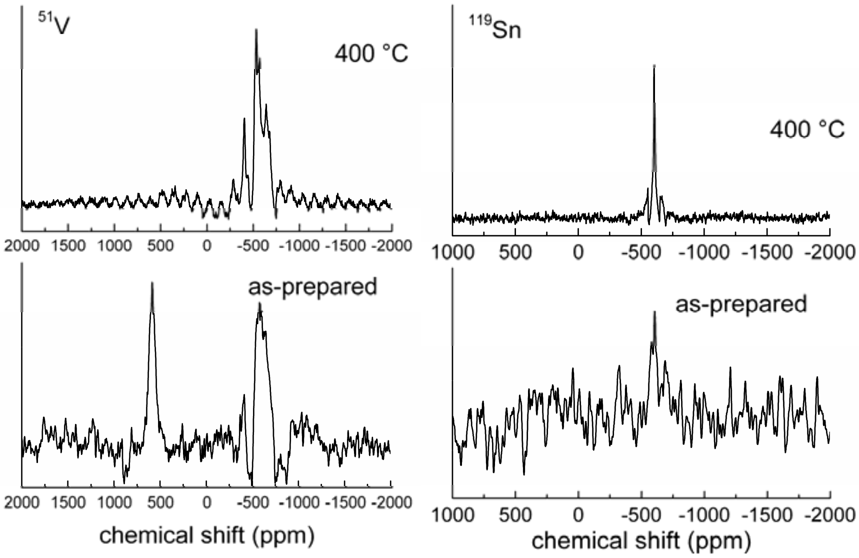

Figure S8: ^{51}V and ^{119}Sn MAS-NMR spectra measured on the as-prepared and 400 °C SnO_2-V_2O_5.

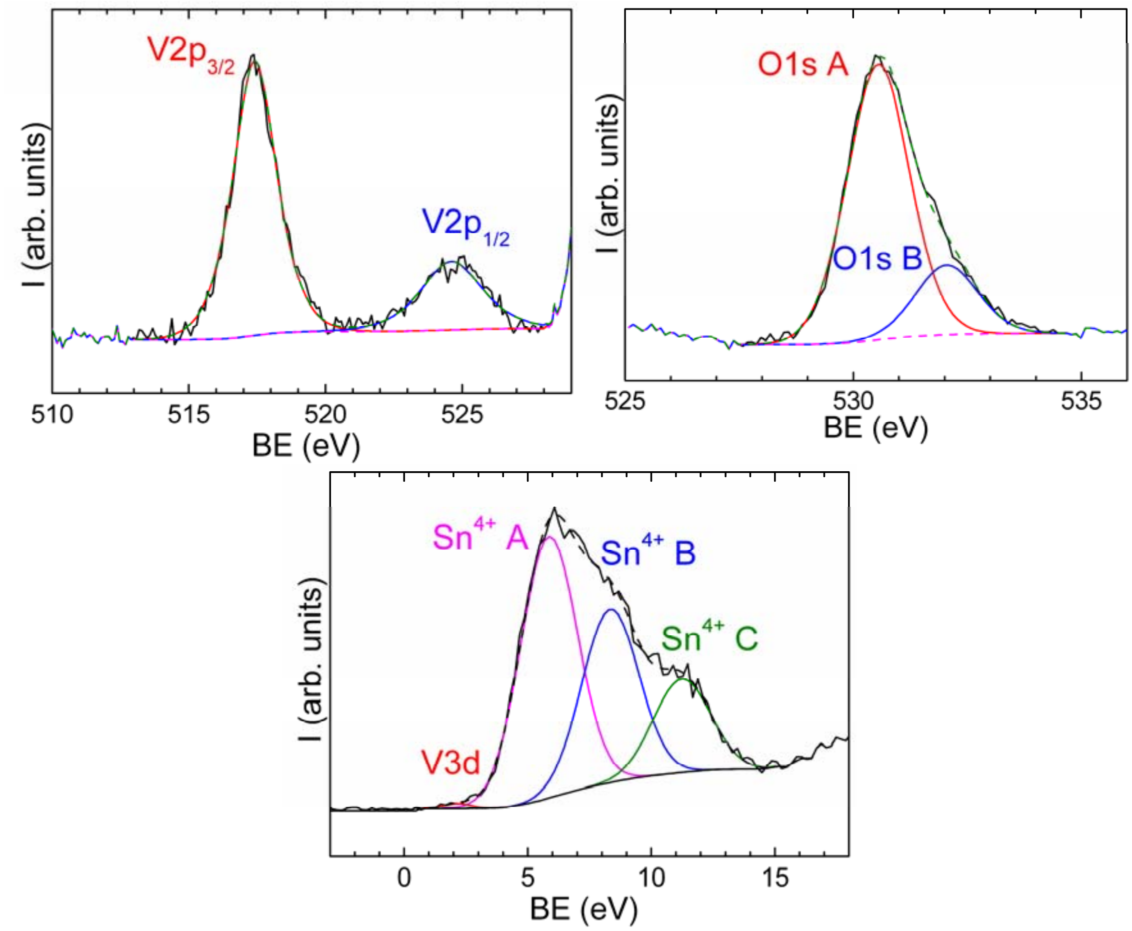

Figure S9: V2p, O1s and the valence band XPS spectra of the 500 °C SnO_2-V_2O_5 sample.

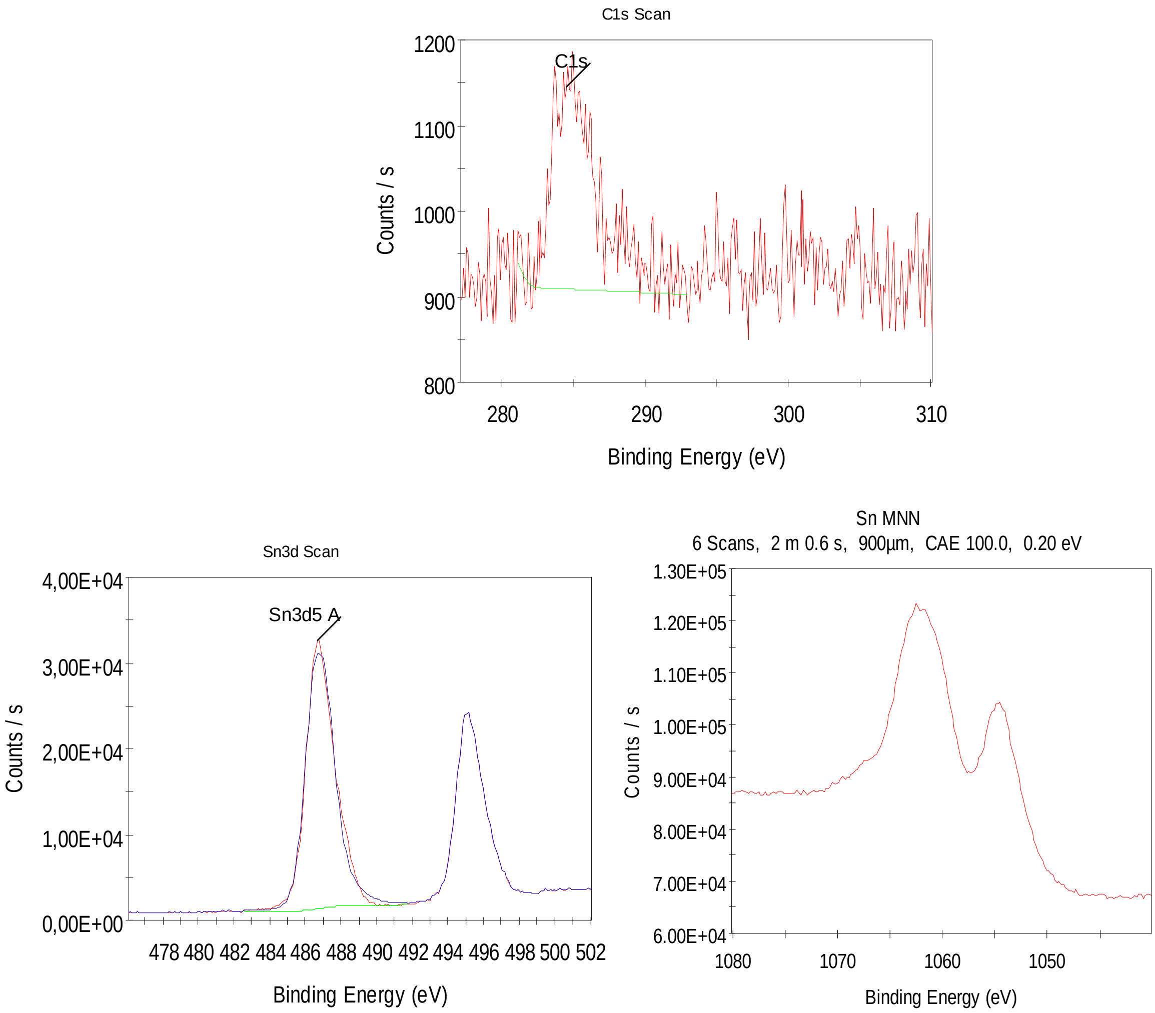

Figure S10: C1s, Sn3d and Sn Auger spectra measured onto the SnO_2-V_2O_5 sample heat-treated at 500 °C.

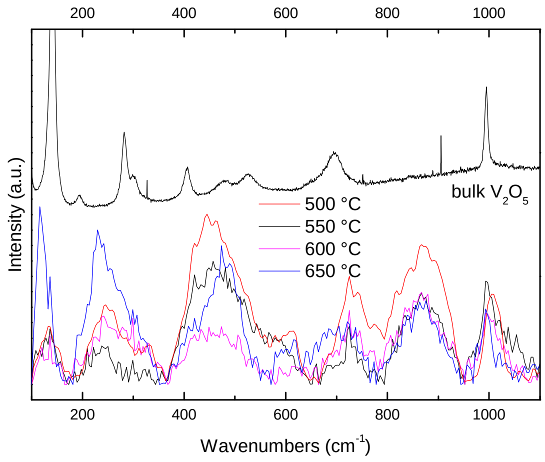

Figure S11: Raman spectra measured on the SnO_2-V_2O_5 samples heat-treated at the indicated temperatures, and spectrum of bulk V_2O_5.

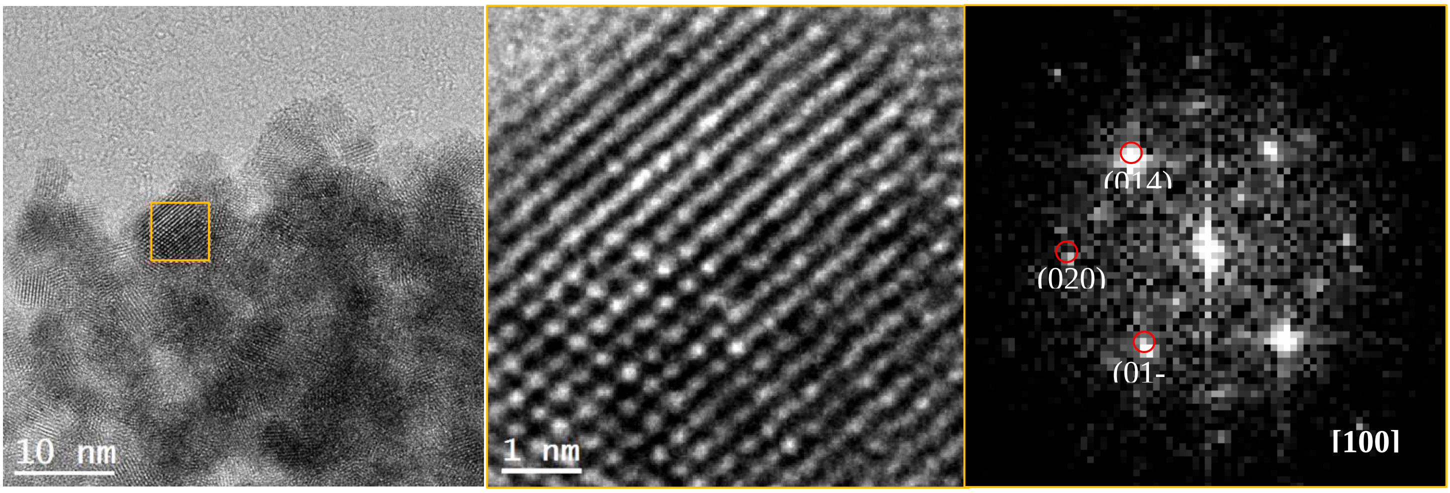

Figure S12: HR-TEM micrograph of the 500 °C SnO_2-V_2O_5 sample. The FFT of selected area marked with yellow square reveals that the nanoparticle can be indexed to the SnO_2 Pbcn, with lattice parameters of a = 0.4737 nm, b = 0.5708 nm and c = 1.5865 nm, orthorhombic (60).

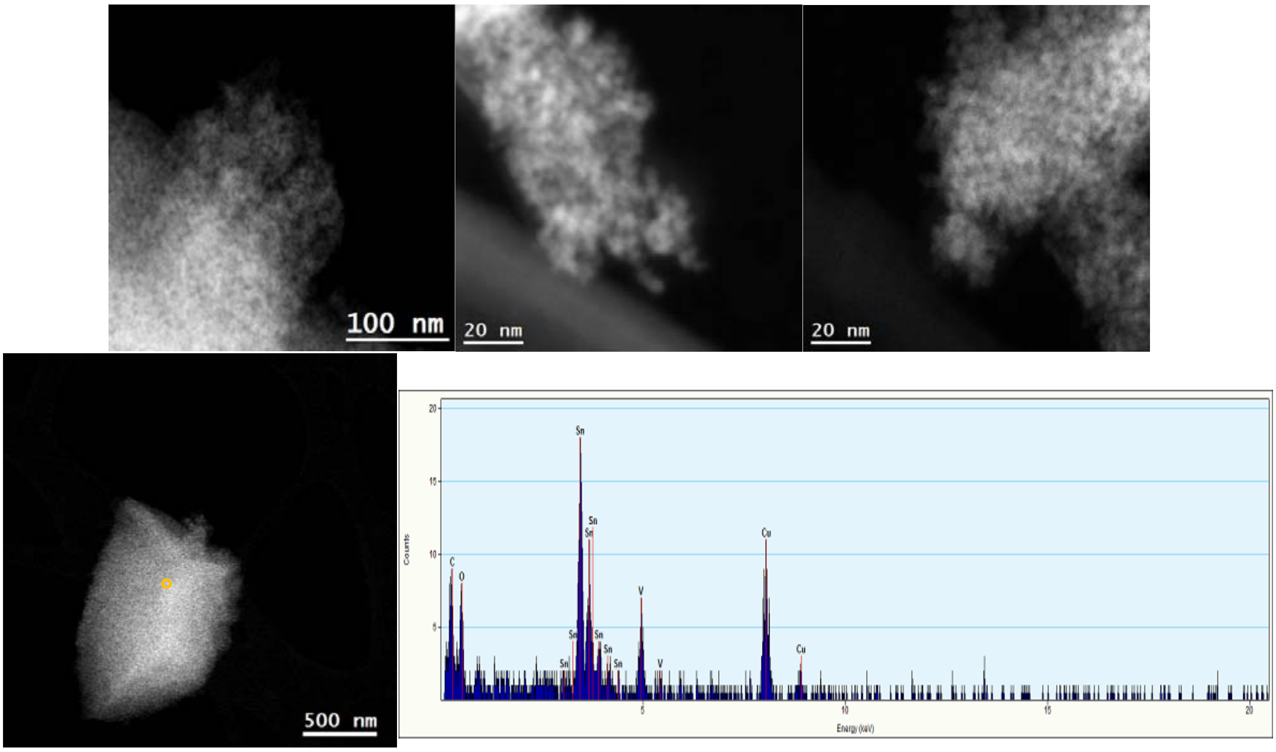

Figure S13: HAADF images (top and left bottom) and the EDX point spectrum of the 500 °C SnO_2-V_2O_5 sample obtained in the yellow point area of the HAADF image (left bottom). Individual Sn, V and O element signals from the sample are present and the C, Cu signal from the lacey C grid.

Thermal behavior of the materials

Thermal analyses were used for optimizing the thermal treatment temperature needed for sample purification from organics, at the same time trying to use the lowest possible temperature for hindering grain growth. The results are shown in **Figure S14**. The TG curve shows a simple structure, with a steep mass loss at about 350 °C. It corresponds to an exothermic region in the DSC curve. This region was obviously associated with the desorption/decomposition of the oleic acid ligands. The mass loss continued slowly after 400 °C up to 650 °C. Since the melting point of bulk V_2O_5 is 690 °C, this mass loss may likely be due to evaporation of the surface vanadium oxide species. In fact, it will be noted from the data in Figure 5 of the paper that the mean size of the SnO_2 nanocrystals in the SnO_2-V_2O_5 samples suddenly grew after heating at 650 °C. This result can hence be seen as further confirmation of the surface modification by the V_2O_5-like layers. For corroborating the thermal analysis interpretation, FTIR spectra were measured onto the corresponding samples. The results are also shown in **Figure S14**. The alkyl and specific oleic acid bands observed in the as-prepared sample disappear after heat-treatment for 1 h at 400 °C.

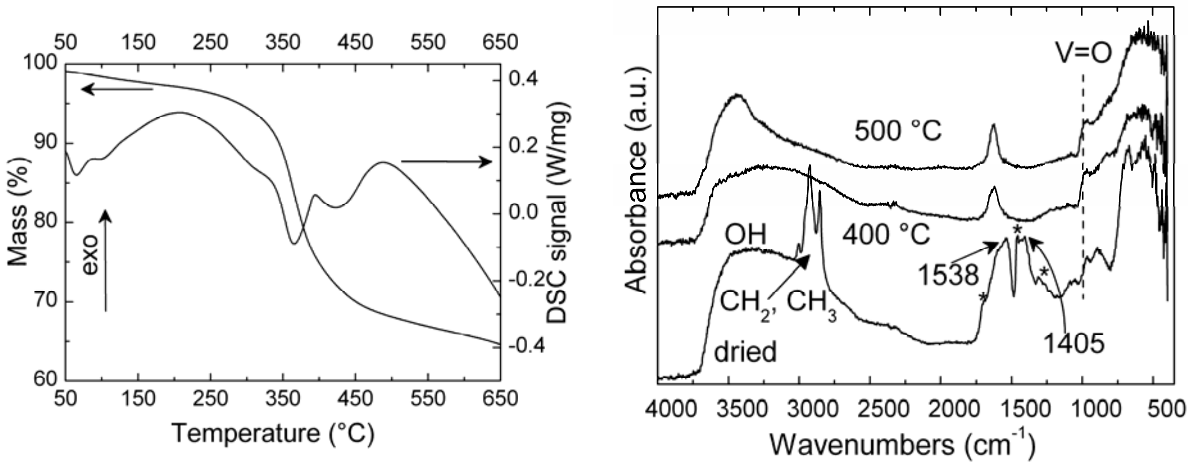

Figure S14: DSC/TG (left) and FTIR (right) spectra measured onto the indicated SnO_2-V_2O_5 samples. The peaks marked with an asterisk and the 1538 cm^{-1} and 1405 cm^{-1} peaks are due to free oleic acid and bonded oleate ions, respectively, and are discussed in the text.

After heat-treatment at 500 °C the only observed change was the narrowing of the OH band, probably due to ongoing condensation reaction in the hydrolytically prepare materials. It can be noted that a band at about 990 cm^{-1} is present even in the high temperature samples. It is typical of vanadyl stretching, and the other V_2O_5 bands were not observed, indicating no phase segregation, in agreement with the previous discussion. For completing the IR discussion, we note the bands marked with an asterisk, due to free oleic acid, and those at 1538 and 1405 cm^{-1}, typical of the asymmetric and symmetric stretching mode of the bonded oleate ions. Their separation, Δ, suggests oleate chelating coordination to the nanocrystal surface.[1-4] From the crossed use of these results and of the XPS data, 500 °C was chosen as the final treatment temperature. As demonstrated until now, this temperature did not result in any vanadium oxide phase separation.

1. Young, A. G.; Al-Salim, N.; Green, D. P.; McQuillan, A. J. Attenuated Total Reflection Infrared Studies of Oleate and Trioctylphosphine Oxide Ligand Adsorption and Exchange Reactions on CdS Quantum Dot Films. *Langmuir* **2008,** *24,* 3841-3849.

2. Yu, W. W.; Wang, Y. A.; Peng, X. G. Formation and Stability of Size-, Shape-, and Structure-Controlled CdTe Nanocrystals: Ligand Effects on Monomers and Nanocrystals. *Chem. Mater.* **2003,** *15*, 4300-4308.

3. Lee, D. H.; Condrate, R. A. FTIR Spectral Characterization of Thin Film Coatings of Oleic Acid on Glasses: I. Coatings on Glasses from Ethyl Alcohol. *J. Mater. Sci.* **1999,** *34*, 139-146.

4. Deacon, G. B.; Phillips, R. J. Relationships between the Carbon-Oxygen Stretching Frequencies of Carboxylato Complexes and the Type of Carboxylate Coordination. *Coord. Chem. Rev.* **1980,** *33*, 227-250.

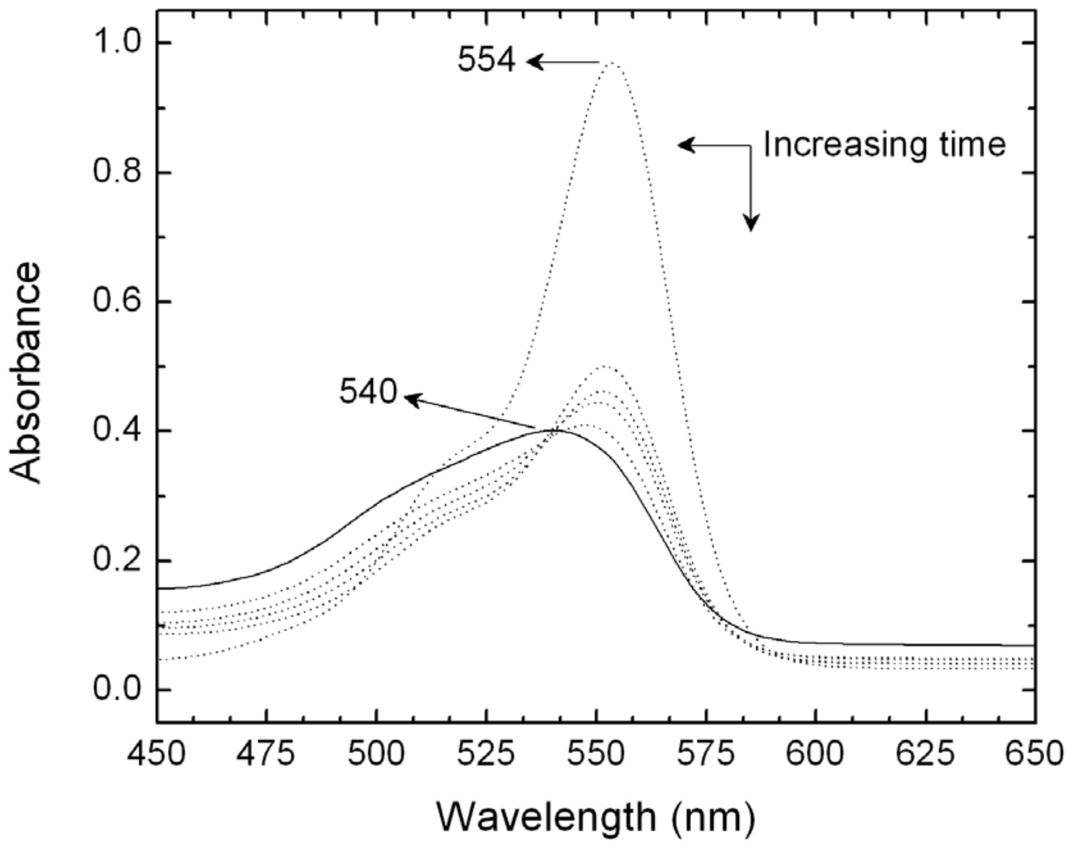

Figure S15: absorption spectra of RhB in the reaction mixture containing the 500°C sample. Solid line refers to mixture spectrum after 60 min.
